# The Effect of Self-Disclosure Skill Training on Communication Patterns of Referred Couples to Counseling Clinics

**Published:** 2014

**Authors:** Eghbal Zarei, Mehri Sanaeimanesh

**Affiliations:** 1 Assistant Professor, Department of Psychology, School of Psychology, Hormozgan University, Bandar Abbas, Iran.; 2 Assistant Professor, Family Counselor, Department of Psychology, School of Psychology, Hormozgan University, Bandar Abbas, Iran

**Keywords:** Communication Patterns, Couple Relationships, Self-Disclosure, Skill Training

## Abstract

**Objective:** This study aimed to examine the effect of self-disclosure skill training on communication patterns of referred couples to counseling clinics in Bandar Abbas.

**Methods:** The applied research design was an experimental study using pre-test and post-test, which was performed on a population of all referred couples to counseling clinics in Bandar Abbas who were interested to participate in a self-disclosure training workshop in response to the announcement. This study was performed on 26 couples who were selected by simple, convenient sampling method; however, they were randomly assigned to the control and experiment groups. A pre-test was administrated before self-disclosure training. The applied instrument includes Christensen and Salavy’s scale of communication patterns. Participants in the experiment group had six sessions of training workshop, each lasted for 90 min. After the intervention, both groups answered the questionnaire again. The collected data were analyzed with paired t-test and covariance statistics.

**Results: **The results showed that the intervention led to significant (p < 0.05) increase in mutual constructive communication pattern and a reduction in mutual avoidance, demand/withdraw, demanding man/withdrawing woman communication patterns. It was also found that the training was not effective on the communication pattern of demanding woman/withdrawing man.

**Conclusion:** The training of simple, but important skills of self-disclosure can help couples to improve their communication and consequently improve their marital satisfaction.

## Introduction

There are different factors that make tension and dissatisfaction in marital life, the most common complaint that help-seeking couples declare are communication problems (1). Adjusting interpersonal relationship is a critical basis for a successful marital life; the situation also can be counted a facilitative process to a stable and satisfying marriage (2).

Family can be seen from several views; one of the best views is the communication channels of interaction among family members, especially couples. Communication patterns are the communication channels, which occur in a family, frequently (3).

One of the important aims of studies in family and marriage fields is to compare the successful couples and unsuccessful couples in terms of communication patterns. Several communication patterns were known, however in this study Christensen and Salavy pattern is chosen as a basis for the current study. They divided the inter-marital pattern of communication into three groups: mutual constructive communication pattern, mutual avoidance communication pattern, and demand/withdrawal (4).

Mutual constructive communication pattern is a type of communication in which both of couples try to discuss about their problem during the process, express their feeling toward each other and suggest a solution for it (5). Heavey et al. explain hat in mutual avoidance communication pattern couples not only avoid talking about problems that they face, but also they try to hide it (5). In the demand/withdrawal communication pattern one of the couples insists on discussing about problems from different views. However the other one avoids the process either physically or psychologically (6). The pattern makes more tension in the marital relationship, as the avoidant member sends a message of indifference toward his/her partner in terms of making a significant interaction (7). The pattern has two subtypes: demand man/withdrawal woman and demand woman/withdrawal man.

Accordingly, couples need to create a normal communication pattern to a set of skills and behaviors which help them to achieve their aims. The behaviors include communication skills in which self-disclosure is one of the most important components. This skill shows how people can express their identity, position, the preferred behavior in an explicit and respectful manner. The skill includes having decisiveness in relationship, and its core is the ability to disclose a person’s thoughts, feeling, interests, experiences, and views toward others (8). In the other words, self-disclosure means what, where, and how many people talk about themselves to the others (9).

There is not a unique definition about self-disclosure. While Jourard defines it as a process of making the -self-known to others, disclosing a set of comments that person express to others, are known as worthy believes (10). Self-disclosure makes life more intimate, emotive, and moral. People’s facial and body expressions will disclose something about them even though they do not talk about themselves (11).

The best well-known theory in this regard is Social Penetration of Altman and Taylor Theory (2). Penetration occurs when the more intimate thoughts and feelings of the discloser begin to be declared to the other person through the relationship. The researchers believe that firstly, engaged people in a relationship disclose some narrow information about themselves, however the trend is cumulative, and the degree of self-disclosure will increase over time, so that in this situation the extent of disclosed information became increased, so that including more private fields of their life (12).

Altman and Taylor used the analogy of self-disclosure as an onion. It means an onion shows two important dimension breadth and depth (12). An onion has many layers in which inner ones represent depth, similar to some details of private life, feeling and thought of people. The external layers of an onion represent the breadth, which includes biographic interests, aims, beliefs, etc. The breadth dimension refers to the frequency or a set of issues that are disclosed by people (13).

At the beginning of the relationship, self-disclosure is also reciprocal. What Altman and Taylor mean by reciprocal is that self-disclosure is high in both individuals at the beginning of a relationship. According to this theory, being willing to reciprocate disclosure allows people to test successively deeper levels of intimacy and thereby to build trust in incremental steps over time (12). Responsiveness and warmth are also important because when people disclose, they want their partners to demonstrate sympathy, understanding, support, and respect for them (13). When a partner is warm, the individual is more likely to disclose again. A warm, responsive partner is likely to disclose as well. When disclosure and responsiveness are not hindered, interactions tend to become increasingly more intimate (14).

It should be considered that too self-disclosure and also lack of self-disclosure lead to serious problems. Some psychologists believe the people who do not talk about themselves prevent affection development as well as interpersonal relationship. Even some psychologists believe psychological problem among people can be ascribed to low levels of self-disclosure (10). However, honesty and self-disclosure should not be mistaken with impulsiveness. Couples should be careful to express all their feelings. To being an honest or reliable person, there is no need to disclose all the feelings (15). Two researchers in a study found that in some limited situations, lack of self-disclosure or confidentiality has a positive correlation with marital satisfaction. There are some problems in the marital life, in which withdrawing from discussion about them is better (16). Some of researcher identified four risks associated with self-disclosure: rejection; reduction of personal autonomy and integrity; loss of control or self-efficacy; and hurting or embarrassing the listener (16).

Despite the importance of self-disclosure to attain healthy and successful marital relationships, there is insufficient evidence regarding b applied researches in the field of self-disclosure training in Iran. Hence, in this research, researchers intend to train the proper method of self-disclosure, and also to compensate the shortages of applied researches by providing more practical model to increase the proper self-disclosure.

## Materials and Methods

The applied design of this study was an experimental method with pre-test and post-test for two times, before and after intervention. There were two groups, the experimental and control in the study.

Study population of this study included all referred couples to counseling clinics in Bandar Abbas who were interested to participate in a self-disclosure training workshop in response to the announcement. From a total of 35 couples registered in the workshop, 30 couples were randomly selected. They were randomly allocated into two groups. The two groups were matched regarding age, the duration of marriage, socio-economic status, education, and the number of family members. Experiment group (13 subjects) received intervention, but the control group (13 subjects) did not. During the study, four couples withdrew from the study, so that finally only 26 couple remained for analysis. The experimental group received six sessions of groups (team) training, which were held weekly for 6 weeks in which each session lasted 90 min. Post-test was administered in the final session and at the end of training. Intervention was directed by a counselor, and the questionnaire was filled out by participants. Researchers provide done educative brochure about self-disclosure skills for the control group to increase their adherence to complete questionnaire forms at the end of their contribution to the study. The content of the training is included in table 1.

In this experimental study, researchers used demographic and communication patterns questionnaires (CPQs). Demographic questionnaire investigated age, the duration of marriage, socio-economic status, education, and the family population.

The CPQ is designed by Christensen and Salavy at California University to study reciprocal communication patterns of couples. It has 35-item and its scale covers a continuum from “1” (not at all) to “9” (yes, often). The questionnaire has three subscales: mutual constructive communication mutual avoidance communication and demand/withdraw communication. The third pattern has two components of demanding man/withdrawing woman and demanding woman/withdrawing man. The researchers assessed the validity of the scale in a sample of couples with three subgroups of “in the processes of divorce," “helpless”, and “normal”. Mutual constructive subscale could determine all three subgroups; however mutual avoidance, and demand/withdraw subscales could recognize the helplessness and normal groups (4).

**Table 1:** Content of the intervention

**Session**	**Content**
**1**	Introducing the leader and members to each other, description of rule and objective of the workshop, definition of self-disclosure, giving homework and pre-test
**2**	An overview of the previous session, training of self-disclosure levels and there levels of stereotype, reality and believes
**3**	Overview, self-disclosure training in four levels of hopes and wishes, feelings, defectiveness, fears and failures, right needs, home work
**4**	Overview, a description of importance of reciprocal, self-disclosure and the partners role in the process, talking training in terms of disclosure and home work
**5**	Overview, training of empathic listening, homework
**6**	Overview, training of perseveration and generation of skills. Conclusion, correcting mistake and post-test

Persian version of this questionnaire has been provided by Ebadatpour. Ebadatpour found Cronbach’s alpha coefficient as 0.70, 0.71, 0.66, 0.51, and 0.52 in for mutual constructive, mutual avoidance, demand/withdraw, demanding man/ withdrawing woman, and demanding woman/ withdrawing man subscales; respectively. The validity coefficient for the questionnaire was counted by concurrent validity of another scale, marital satisfaction results showed a very good coefficient between the components (17). Researchers in this study measured reliability coefficients via Cronbach’s alpha as: 0.76, 0.91, 0.79, 0.58, 0.76, and 0.70 for its components as the above mentioned order.

Data are expressed as means ± standard deviation. To confirm the mean difference between the control and experimental groups a paired t-test was performed (Tables 2 and 3). Furthermore, researchers used analysis of covariance (ANCOVA). p < 0.05 was considered to be statistically significant.

## Results

According to table 2, no significant differences were observed in the controls between pre-test and post-test in term of couples’ communication patterns.

According to table 3, all the communication patterns components expect demanding woman/withdrawing man were significant in the experiment group.

One of the assumptions for ANCOVA is the homogeneity of error variances, which is tested by Levene’s test. Levene’s test showed: F = 0.019, df = 1 and 50, p = 0.890; thus the assumption was not violated and the variances of both groups were equal.

The other assumption to test the ANCOVA is the examination of the regression of homogeneity slip. Results of table 4 showed that contrast impact of pre-test and group on the dependent variable is not significant in alpha level of 0.1. Therefore, not being of significance in this interaction approves the assumption of regression slop homogeneity.

**Table 2:** Paired t-test for the control group (pre-test and post-test). Data are showed as means ± standard deviation. No significant differences were observed regarding couples’ communication patterns

**Communication patterns**	**Control**	**Mean (**±**SD**†**)**	**t**	**P-value**
**Mutual constructive**	Pre-test	17.50 (±2.81)	-0.40	0.69
	Post-test	17.84 (±2.89)		
**Mutual avoidance**	Pre-test	09.11 (±2.81)	-0.38	0.70
	Post-test	08.73 (±2.89)		
**Demand/withdraw communication**	Pre-test	08.61 (±1.57)	-1.02	0.31
	Post-test	09.11 (±1.77)		
**Demand man/withdraw woman**	Pre-test	14.88 (±4.28)	-0.44	0.65
	Post-test	15.30 (±3.38)		
**Demand woman/withdraw man**	Pre-test	14.96 (±3.46)	-0.32	0.74
	Post-test	15.34 (±2.28)		

† Standard deviation

**Table 3:** Paired t-test the experimental groups (pre-test and post-test). Data are displayed as means ± standard deviation. Significant differences were observed regarding couples’ communication patterns except for demanding woman/withdrawing man

**Communication patterns**	**Experiment**	**Mean (**±**SD**†**)**	**t**	**P-value**
**Mutual constructive**	Pre-test	17.60 (±2.82)	-21.90	0.001
	Post-test	20.20 (±1.81)		
**Mutual avoidance**	Pre-test	08.96 (±1.90)	-03.36	0.002
	Post-test	07.53 (±2.24)		
**Demand/withdraw communication**	Pre-test	09.11 (±1.77)	0-2.37	0.020
	Post-test	10.30 (±1.25)		
**Demand man/withdraw woman**	Pre-test	15.38 (±3.38)	-05.94	0.001
	Post-test	10.19 (±2.60)		
**Demand woman/withdraw man**	Pre-test	15.34 (±3.38)	-00.37	0.690

† Standard deviation

**Table 4:** The analysis of homogeneity of regression slip of couples communication patterns

**Dependent variable**	**Source of change**	**Sum of squares**	**df** †	**Means of squares**	**F**	**P-value**
**Couples communication patterns**	Group	019.48	01	19.48	2.3	0.1
	Pre-test	000.96	01	00.96	0.1	0.7
	Reciprocal effect	022.51	01	22.51	2.7	0.1
	Error	405.50	48	08.44		
	Total	0017,461	52			

† Degree of freedom

**Figure 1:**
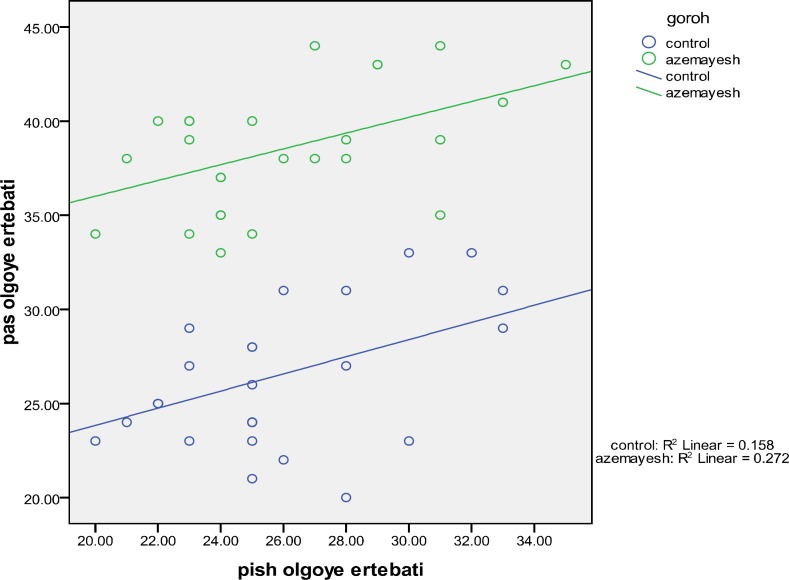
Scoter plot for pre-test and post-test based on groups

**Table 5:** Analysis of covariance for communication patterns shows that all the communication patterns expect demanding woman/withdrawing man were significant

**Communication patterns**	**SS** †	**df** ‡	**MS** §	**F**	**P**	**ETA** || ** coefficient**
**Mutual constructive**	1524.00	1	1524.00	201.70	0.01	0.800
**Mutual avoidance**	0027.96	1	0028.00	005.79	0.02	0.100
**Demand/withdraw communication**	0021.40	1	0021.40	009.37	0.01	0.160
**Demand man/withdraw woman**	0331.90	1	0332.00	038.65	0.01	0.380
**Demand woman/withdraw man**	0002.99	1	0002.99	000.34	0.56	0.007

† Sum of squares;

‡ Degree of freedom;

§ Mean of square;

|| Parital Eta square

Based on figure 1, regression line of control and test groups are parallel and this shows the existence of linear relationship between dependent variable and auxiliary random variable.

According to table 5, all the communication patterns expect demanding woman/withdrawing man were significant.

## Discussion

According to findings of this study self-disclosure training was effective for all components of the communication patterns expect demanding woman/withdrawing man. No similar studies investigating the aim of this study were found. Applying the training of self- disclosure in relation to communication patterns showed novelty and exploratory properties of this research by its considerable findings. So that we observed that self-disclosure training increased the mutual constructive communication pattern and reduced the mutual avoidance, demand/withdraw, and demanding man/withdrawing woman communication patterns. Evidence implies that communication skills play a major role in communication patterns. As the statistical findings showed in the mutual constructive communication pattern, training the self-disclosure skill have had impact on the experimental group. It can be said that this training intervention regarding self-disclosure helps couples to create a more healthy relationship together, to express more empathy and more perception through consciously conversations, and also to create a safe environment to satisfy the needs. Through the learning of consciously conversation skill, couples can interchange information of themselves and their spouse in the right way and can perceive the needs and vulnerabilities of each other. 

About mutual avoidance communication pattern can be said that avoidant couples do not have a special method to solve their conflicts so that they expect that problems to be solved over time. Familiarizing them with the communication skills help them to have better management in their relationships. Training the communication skills help people to use their emotions, thoughts and other mental states to overcome on inefficient communication patterns.

Another finding of this study was that training of the self-disclosure skill has had an impact on the demand/withdraw communication pattern of experimental group. These findings explain that couples that fall in negative interactions do not have a logical and clear approach to exit from this incorrect cycle. In fact, training of communication skills might help them to exchange their messages with more accuracy. Systematic practice of these skills bring about people add this habit to their behavior collection.

In a study researchers compared two training programs to improve communication patterns and marital adaption among 131 couples (1). This study indicated the couples who participated in communication skills training session experienced increasing positive feelings toward partner (1). Rimaz studied the effectiveness of communication skills training to change communication patterns in Naghde, Iran. The results showed a positive change in communication pattern of the couples (18).

In another study researchers trained the communicational skills with cognitive-behavioral approach to 67 couples that had registered for education of communication through newspapers call. After educating, couples used better communication patterns (1).

As the statistical analysis of demand woman/withdraw man communication pattern showed the self-disclosure skill did not influenced the experimental group. It might be a biased conclusion because among dissatisfied couples, wives feel their husbands are avoider though their husbands state their wives are engaged in conflict so much. Some of researcher explained that the withdrawing husbands often try to increase the level of hostility with their wives, so that this pattern was destructive for the marital relationship (19) There are two structures for conflict and individual differences between two genders which explains this finding. In the conflict structure perspective the higher status and power typically granted to men, which encourages them to avoid conflict because they have no interest in change. On the other hand, women typically have less power and see conflict engagement as a means of obtaining what they want. That is, the larger social structure, which gives men greater power and women less power, leads to women having more investment in change than men. As such, people in the demanding role generally want more closeness, while those in the withdrawing role want more distance or autonomy (20).

The individual differences perspective is mostly based on the findings of Gottman and Levenson that, in which men experience more physiological arousal during conflict than women. They suggest that men’s higher level of physiological reactivity leads them to try to escape from the unpleasant feeling, whereas women, being less physiologically reactive to stress, do not desire to avoid conflict (20). Altogether it can be said the best way to improve the communication patterns is adding other types of communication skills to self-disclosure skills.

Ahmadi et al. (21) assessed the effectiveness of a shorter term couple therapy on couples’ communication patterns. Findings indicated that the interaction was significantly effective for all components of the communication pattern expect mutual avoidance pattern (21).

Communicational problems in marital relations are amongst the most important factors that influence the dissatisfaction of couples regarding marital life. Communicational problems are due to lack of proper communicational skills of couples. Hence, training of simple but important skills regarding self-disclosure, can help them to improve the communication and thus to increase the satisfaction of marital relationship. The results of a research showed couples that share their thoughts easily and are able to accept and understand each other’s feelings have more satisfaction (20).

In this study, it has been tried to move from external layers of personality towards internal ones via training self-disclosure skills. It was proposed that couple will be more intimate by exchanging their private information. After changing the massages into feeling, the couple can understand core characteristics of their personality. Then they can move to internal layers, so that with reciprocal self-disclosure their massages become deeper and widener.

The training of simple but important skills of self-disclosure can help couples to improve their communication and consequently improve their marital satisfaction.

Small sample size and no longer follow-up sessions (with re-testing) were the major limitations of this study. 

## Notes

**Declaration of interest:** None.
